# Assess, improve, detect, guide: a narrative review and proposal for a standardized protocol for prehospital transesophageal echocardiography during out-of-hospital cardiac arrest

**DOI:** 10.1186/s13089-025-00418-4

**Published:** 2025-03-05

**Authors:** Stephan Katzenschlager, Thomas Hamp, Maximilian Dietrich, Christopher T. Edmunds, Nikolai Kaltschmidt, Markus A. Weigand, Mario Krammel, Frank Weilbacher, Erik Popp

**Affiliations:** 1 Medical Faculty Heidelberg, Department of Anesthesiology, Heidelberg University, Im Neuenheimer Feld 420, 69120 Heidelberg, Germany; 2 Emergency Medical Service Vienna, Vienna, Austria; 3 Department of Anesthesia, Intensive Care Medicine and Pain Medicine, Medical University of Vienna, Vienna, Austria; 4 Department of Research, Audit, Innovation, and Development, East Anglian Air Ambulance, Gambling Close, Norwich Airport, Norwich, NR6 6EG UK; 5 University of East Anglia, Norwich, UK; 6 Critical Care, North West Anglian Foundation Trust, Peterborough, UK; 7 PULS Austrian Cardiac Arrest Awareness Association, Vienna, Austria

**Keywords:** Transoesophageal echocardiography, Out-of-hospital cardiac arrest, Advanced life support, Prehospital, Emergency medical service

## Abstract

While there are different protocols for in-hospital transesophageal echocardiography, there is no existing protocol for prehospital usage during out-of-hospital cardiac arrest. Herein, the “assess-improve-detect-guide” protocol is described. This protocol includes four mid-esophageal views to address the most time-sensitive aspects during out-of-hospital cardiac arrest. This protocol can be used in services with and without the capability for eCPR, with the benefit of profiting from additional diagnostic and personal resources. This protocol provides a standardized approach for performing transesophageal echocardiography during out-of-hospital cardiac arrest and reporting the results.

**Supplementary Information:**

The online version contains supplementary material available at 10.1186/s13089-025-00418-4.

## Background

Ultrasound has become more available in the past decades, and since 2015, it has been recommended to detect reversible causes of cardiac arrest [1]. Different protocols for transthoracic echocardiography (TTE) during out-of-hospital cardiac arrest (OHCA) are in place [2, 3]. Although not specified in current guidelines, TTE should be performed during rhythm checks to obtain the best possible images and limit chest compression interruptions. Consideration should be made when interpreting the images outside a rhythm check [1]. However, TTE views can be limited due to gastric inflation, defibrillation pads, and hand positioning, potentially resulting in the inability to use this technique.

Further, extensive experience is necessary to obtain the desired images [1]. In comparison, transesophageal echocardiography (TEE) helped to detect reversible causes and the area of maximal compression (AMC) during emergency department treatment (ED) of OHCA [4–6]. In cases of uncertainty, TEE can help to characterize fine ventricular fibrillation [7]. Additionally, pulse checks were shortest with TEE compared to TTE and manual pulse checks [8]. TEE can facilitate goal-directed resuscitation with fluids, blood products, or inotropes [9]. In the absence of fluoroscopy, TEE has shown to be reliable during extracorporeal membrane oxygenation (ECMO) placement with a 100% success rate and without the need for repositioning [10].

Case reports have used prehospital TEE during OHCA [11, 12]. The feasibility was demonstrated in a small cohort study, where the time until the first image was acquired was five minutes, starting with the research team entering the scene until a mid-esophageal four-chamber view was obtained [13]. While there are protocols for ED TEE in place [4], these have yet to be described for prehospital care as this is a resource-depleted setting with time constraints. Usually, there is no provider on the scene capable of performing a TEE, which implies that a specialized enhanced care team with an experienced TEE provider must be dispatched.

Herein, we aim to provide a comprehensive protocol and rationale for using prehospital TEE during OHCA, combined with standard advanced life support (ALS) or extracorporeal cardiopulmonary resuscitation (eCPR). Such a protocol is necessary to provide emergency medical teams with a structured approach.

## Methods

We conducted a narrative literature review to understand where in-hospital experience might be translated to pre-hospital practices. Search terms for PubMed were “cardiopulmonary resuscitation”, “out-of-hospital cardiac arrest”, “echocardiography”, and “ultrasound”.

Additionally, the practical experience of the author's advanced prehospital response team was used to provide a comprehensive TEE protocol with a focus on adult non-trauma OHCA.

### Ultrasound devices

Prehospital TEE can be performed with different portable devices. However, it must be clarified that these devices are not CE-certified for prehospital use. There is no approved holder for transportation in an ambulance. Ethics committees can determine if the usage is appropriate during clinical studies, as these devices are certified for a stationary in-hospital setting in critical patients.

Current devices are portable, weigh between 3.5 and 10.5 kg, and have a one to two hours battery life.

### In-hospital protocols

The literature demonstrates the use of TEE well in emergency department settings. Clinical guidelines and protocols are essential to ensure that the operating clinician answers diagnostic questions in a rapid and targeted fashion. The accepted practice is to use either a complete or rapid protocol for cardiac assessment [14, 15]. The situation usually dictates the level of evaluation completed. Several protocols have been described for specific circumstances; the most relevant are those for intra-cardiac arrest assessment.

The windows used in almost all protocols are like those in transthoracic echocardiography except for the ME bicaval view. TEE windows utilized are mid-esophageal 4 chambers, mid-esophageal long axis, transgastric short axis, and mid-esophageal bicaval [4, 8, 16]. There is no literature to support any specific protocol; however, it is recognized that targeted image acquisition is crucial [17]. This has been identified as a critical issue in the pre-hospital setting, where diagnostic findings are evolving, and environmental challenges mean images are technically more challenging.

## Proposed prehospital protocol

**Fig. 1:**
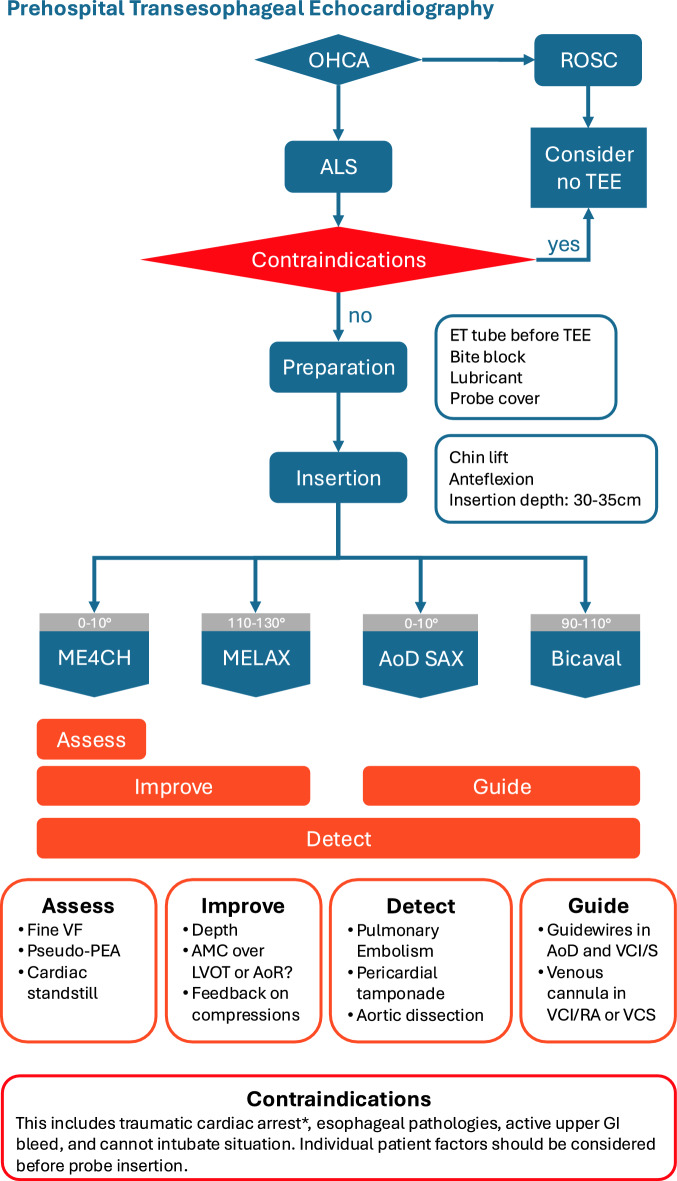
Prehospital Transesophageal Echocardiography protocol. *Traumatic cardiac arrest is a relative contraindication. OHCA, out-of-hospital cardiac arrest; ROSC, return of spontaneous circulation; ALS, advanced life support; TEE, transesophageal echocardiography; ET, endotracheal; ME4CH, Mid-esophageal four-chamber; MELAX, Mid-esophageal long axis; AoD SAX, Mid-esophageal Descending aortic short-axis; VF, ventricular fibrillation; PEA, pulseless electric activity; AMC, area of maximal compression; LVOT, left ventricular outflow tract; AoR, aortic root; AoD, descending aorta; VCI, vena cava inferior; VCS, vena cava superior; RA, right atrium; GI, gastrointestinal

Figure 1 displays the flowchart for the proposed prehospital TEE protocol.

### Implementation, Logistical challenges, and proposed training level

Depending on the available infrastructure and financial resources, one can purchase disposable probes that eliminate the need for cleaning after each use and help prevent any damage to the probes. Reusable probes have minimal per-use costs but require cleaning after each use, adhering to strict hygiene protocols.

The portable TEE machine can be stored in the ambulance car or at the station. The downside of the latter is that it must be taken with every call and secured for transportation. If stored in the vehicle, it should be easily accessible and always charged [18]. Further, the car's inside temperature should be steady, which is optimal for the TEE machine.

Current guidelines recommend a minimum of 10 TEE examinations on patients and simulators [8]. However, some programs have increased the number of exams needed for in-hospital resuscitative TEE [19]. Due to the time-sensitive nature and complexity of resuscitative TEE, prehospital providers should have profound experience in cardiac anesthesia or critical care echocardiography. One of the most challenging parts of resuscitative TEE is determining the AMC and correctly suggesting changes to the rescuers. This can be achieved through case discussion and case feedback.

There are various options for implementing prehospital TEE. EMS physicians with additional training can facilitate TEE [11, 20], and a dedicated study team [13, 21], or a specialized response team [18, 22] can be dispatched.

### Potential risks, limitations, and contraindications

Potential risks include bleeding, accidental extubation, or probe damage. The most common risk reported with TEE is minor upper gastrointestinal bleeding [23, 24]. To our knowledge, no endotracheal tube dislocations have been reported. Certain studies have excluded persons with known esophageal diseases [13] or after trauma [4].

Recent studies have not reported serious adverse events related to TEE [16–18]. However, these risks can be limited by rigorously using a bite block, adequate tube fixation, and careful probe handling with lubricant.

Impairing chest compression quality should be avoided under all circumstances.

One limitation during OHCA is that optimal views with all structures present can only sometimes be obtained.

Absolute and relative contraindications for prehospital TEE stem from the American Society of Echocardiography guidelines [25]. Absolute contraindications include esophageal pathologies (e.g., stricture, tumor, or perforation), a perforated viscus, and an active upper gastrointestinal bleed [25]. Information on the patient’s history is limited in the prehospital setting; therefore, it cannot be guaranteed that absolute contraindications are followed.

Prehospital-specific contraindications are the inability to endotracheal intubate and traumatic cardiac arrest. The latter can be seen as a relative contraindication due to different treatment priorities.

### Preparation and insertion

During the initial ALS phase, a dedicated team member prepares the necessary equipment for TEE. An endotracheal tube is mandated for all cases. Supraglottic airway devices should be replaced simultaneously with other ALS interventions. Commercial devices, such as the Thomas™ tube holder (Laerdal Medical Corporation, Stavanger, Norway), may have to be replaced to insert the TEE probe. An elastic bandage or adhesive fixation can be used as an alternative.

As mandated by the ALS guidelines, continuous etCO2 monitoring is required to monitor endotracheal tube placement and changes in cardiac output during AMC optimization.

A bite block should be used to minimize probe damage due to signs of life during resuscitation. If an oro- or nasogastric tube is already placed, removing it can help with probe insertion. Minor artifacts due to the oro- or nasogastric tube can reduce image quality and impede assessment. Probe cover and lubricant are considered standard materials.

Before insertion of the probe, contraindications for TEE, as listed above, should be excluded.

Pausing chest compressions is not required for probe insertion. One person should perform a chin-lift maneuver while the operator inserts the TEE probe. The chin-lift maneuver can be released once the probe is inserted to 30-35cm measured at teeth.

### Views

#### ME4CH

**Fig. 2:**
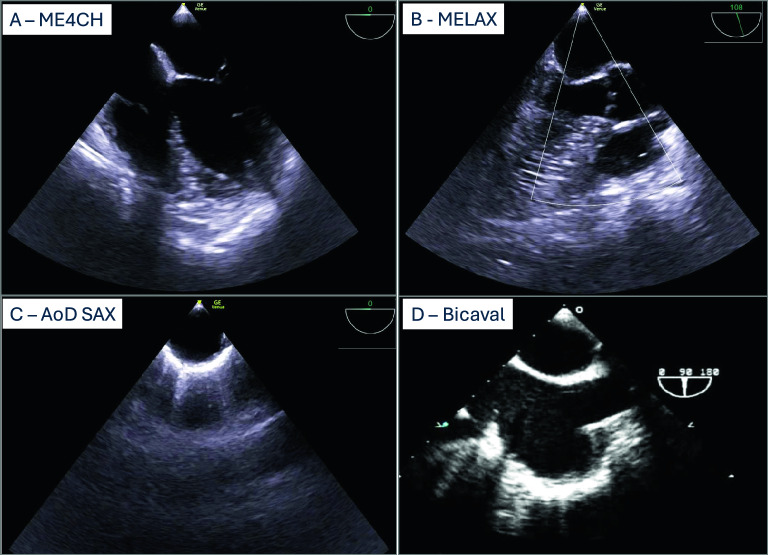
**a**–**d** Prehospital TEE views. **A** Mid-esophageal four-chamber, **B** Mid-esophageal long axis, **C** Mid-esophageal Descending aortic short-axis, **D** Mid-esophageal Bicaval. ME4CH, Mid-esophageal four-chamber; MELAX, Mid-esophageal long axis; AoD SAX, Mid-esophageal Descending aortic short-axis

First, the mid-esophageal four-chamber (ME4CH) view is obtained to demonstrate ventricles and atria. The probe is inserted to 30 to 35cm until the mitral valve is identified. Ideally, the probe is retroflected to minimize foreshortening; however, this should not be the focus during OHCA. The aortic valve should not be visible. By turning the probe, the left or right ventricle can be centered (Fig. 2a).

#### MELAX

The mid-esophageal long axis (MELAX) view is achieved by rotating the probe to 110–130° at the same depth. This second view should visualize the left atrium, left ventricle, left ventricular outflow tract, aortic valve, and ascending aorta. The ascending aorta can be assessed by slightly retracting the probe (Fig. 2b).

#### AoD SAX

As the third view, the descending aorta short axis view (AoD SAX) is displayed by rotating the probe to 0–10° and turning it to the left (counterclockwise) (Fig. 2c). Depth should be adjusted to ensure optimal image quality.

#### Bicaval

In cases of eCPR cannulation, the bicaval view is needed. Turning the probe to the right (clockwise) and rotating forward to 90–110°. In the bicaval view, the right atrium is centered, the superior vena cava is on the right, and the inferior vena cava on the left (Fig. 2d).

### Interventions

This proposed protocol comprises the TEE views mentioned above during OHCA. It aims to potentially assess the underlying rhythm in cases of uncertainties, improve chest compression quality, detect reversible and irreversible causes, and help guide wires and the venous cannula in case of eCPR (Table 1).

**Table 1:** Protocol with focused assessment and included views for prehospital TEE during OHCA

Protocol	Focused assessment	TEE views
Assess	Rhythm analysis Fine VF? Pseudo-PEA?	ME4CH
Improve	Chest compression Depth? Area of maximal compression?	ME4CH MELAX
Detect	(Ir-)reversible causes Pulmonary embolism? Pericardial tamponade? Aortic dissection? Intracardiac thrombus	ME4CH MELAX AoD SAX Bicaval
Guide	Arterial- and venous guidewire, Venous eCPR cannula	AoD SAX Bicaval

VF, ventricular fibrillation; PEA, pulseless electric activity; eCPR, extracorporeal cardiopulmonary resuscitation; ME4CH, mid-esophageal four-chamber; MELAX, mid-esophageal long axis; AoD SAX, descending aorta short axis

Specific interventions based on the findings can be undertaken immediately under continuous TEE guidance. Some patients may have achieved a return of spontaneous circulation (ROSC) before the specialized enhanced care team arrives. In these cases, TEE may be considered after careful risk–benefit evaluation in profoundly unstable patients, considering the urgent transport to the hospital.

#### Assess

Rhythm assessment can be undertaken with the ME4CH view immediately after probe insertion. Fine ventricular fibrillation, not detected by external defibrillator pads, can be seen on TEE and should be treated appropriately [4] (Supplemental Video 1). Pseudo-pulseless electric activity can be detected by using TEE. Combining TEE with an arterial line allows for a better assessment of cardiac output compared to manual palpation and can optimize hemodynamic management.

#### Improve

Chest compression quality assessment via TEE consists of accurate depth and the location of the AMC. Additionally, chest compression rate can be assessed. However, live feedback sensors are recommended by current guidelines [1] and can determine the chest compression rate [26]. Combining the MELAX and ME4CH views allows a comprehensive assessment of the AMC (Supplement Video 2,3). A more caudal compression point should be aimed for if the AMC lies over the aortic root or the left ventricular outflow tract (LVOT). These changes can be made during ongoing TEE assessment to observe the immediate effect. Closure of the left ventricular outflow tract was associated with a higher rate of death after OHCA [5]. In an animal model, chest compression over the LVOT resulted in lower aortic pressure and end-tidal CO_2_ (etCO_2_) [27]; therefore, aiming to improve the AMC with TEE.

Using standardized ventilation, etCO_2_ correlates well with cardiac output and chest compression quality during OHCA [28–30]. Adding to the visual and etCO_2_ assessment during changes in the AMC, arterial blood pressure monitoring can be used to monitor the respective changes. A recent study demonstrated the usage of the M-mode to assess the AMC over the left ventricle or LVOT [27].

#### Detect

Detecting (ir-)reversible causes can guide management during the initial stage of OHCA. Evaluating the size of the right ventricle is feasible in the ME4CH view and provides a first impression of right ventricular performance. Combined with intraatrial or intraventricular thrombus, pulmonary embolism can be proven. Within minutes of cardiac arrest onset, the right ventricle dilates, even when caused by hypovolemia, hyperkalemia, or arrhythmia [31]. An isolated right ventricular dilation cannot definitively indicate pulmonary embolism, nor does the absence of a small right ventricle automatically exclude cardiac arrest due to hemorrhagic shock [32]. Pericardial effusion can be observed due to aortic dissection or after myocardial infarction due to myocardial rupture (Supplemental Video 4) [33]. Extensive hemopericardium combined with an aortic dissection flap in the ascending or descending aorta is highly suggestive of a type-A dissection. The continuation of resuscitation maneuvers may be considered futile in case of cardiac arrest and the absence of cardiac movements. However, it is not recommended to use ultrasound findings alone as a decision tool for terminating resuscitation [34]. This is because the definition of "cardiac movements" in different clinical studies is non-univocal, so further interventions are needed.

#### Guide

To increase the safety of this procedure, arterial and venous guidewires should be visualized after ultrasound-guided access. Both guidewires can be visualized using the bicaval and descending aorta views before dilating the femoral vessels. If a guidewire cannot be seen in the anticipated view, the other view should be checked thoroughly for a double-wire sign. Ideally, the venous guidewire can be seen across the right atrium into the superior vena cava, and the arterial guidewire can be visualized in the descending aorta. This ensures that the desired vessels are cannulated in an error-prone setting. Under continuous chest compressions, the placement of the venous cannula can be guided by TEE. Depending on the drainage cannula, placement can either be at the vena cava inferior/right atrium junction or with the tip in the superior vena cava [35]. For optimal visualization of the vena cava inferior/right atrium junction, the TEE probe must be 1–2 cm advanced.

After successful cannulation and return of circulation, a focused assessment of the cardiac function can be done while the cannulas are being fixated. The ME4CH and MELAX views allow for assessing the left ventricular function and contractility, possible regional wall motion abnormalities, and the aortic valve opening during eCPR. This should not prolong extraction and transfer to the nearest suitable cardiac arrest center. Due to the limited space in an ambulance and the lack of secure storage of the TEE during transport, removing the probe before patient extraction can be considered. Disinfection can be done with a high-level disinfectant foam to reprocess ultrasound probes. Alternatively, in-hospital sterilization is possible; however, a longer turnaround time must be anticipated.

### Preliminary experience

A feasibility study describes the first experiences of prehospital TEE in Vienna [13]. Since 2023, prehospital TEE has been used at Heidelberg University Hospital. A total of 27 cases were recorded, including 13 eCPR cases. Two patients with aortic dissection type A were identified in whom CPR was ceased. Further reversible causes included pericardial tamponade and pulmonary embolism identified through clots in the right atrium. Changes in the AMC were made in 26% (7/27) of cases.

TEE was used in all cases for prehospital eCPR to visualize both guidewires during cannulation. In a few instances, the descending aorta could not be visualized during chest compressions. Weighing the risk vs. benefit, chest compressions were paused for a couple of seconds and resumed after identification of the descending aorta. Another option is to visualize the arterial guidewire in the MELAX view; however, this comes with the drawback that a guidewire entering the extracranial vessels may be overlooked. Ideal positioning of the venous drainage cannula was also possible after initial placement and starting of ECMO flow. Further prehospital assessment after achieving return of circulation is not specified in our institution, allowing for rapid transport to the nearest cardiac arrest center.

## Discussion

This concept paper describes the use of TEE during OHCA in the prehospital field—a focused protocol compatible with time limitations. The “assess-improve-detect-guide” approach helps providers conduct a focused assessment. This proposed protocol lays the foundation for a standardized approach.

Other protocols include the transgastric short-axis or the mid-esophageal right ventricular inflow-outflow tract [4, 8, 16, 19]. These views are feasible in the prehospital setting; however, adding more views into a protocol consumes more time. Additionally, extensive movement of the probe can cause damage to the esophagus. This is especially relevant in the prehospital field, where little is known about prior diseases. This proposed protocol defers the transgastric view to balance feasibility and risk limitation, as relevant pericardial effusion can be seen in the ME4CH view [8]. This proposed protocol includes the AoD SAX for the assessment of aortic pathologies. Combined with evaluating the ascending aorta in the MELAX, one can detect contraindications for eCPR placement [36]. Although survival after aortic dissection and OHCA is reported to be 0% [34, 37], one should be cautious in the absence of pericardial effusion where a chronic type-B dissection is suspected.

When introducing a new tool into a resource-deprived setting, ethical considerations should be made. As part of a clinical trial, informed consent before enrollment is impossible in OHCA [38]. Notification of enrollment, and therefore the use of TEE, should be made as soon as possible [38].

Potential transport delays can limit the chance of other life-saving interventions, such as eCPR or cath lab examinations. For patients with ROSC and those eligible for in-hospital eCPR, this proposed protocol suggests not using TEE in favor of early transportation. Most OHCA patients are in a non-shockable rhythm [39] and, therefore, are ineligible for most eCPR centers [40]. These patients can profit from optimizing ALS on-scene and evaluating potential causes of OHCA. In-hospital surveys found that financial barriers were the most perceived, followed by maintenance, credentialing/privileges, and the lack of TEE-trained physicians [19].

The overarching goal of CPR measures should be rapid treatment of the underlying cause and transport to the nearest cardiac arrest center for further care. Therefore, this protocol is designed to help individualize resuscitation efforts at an early stage.

Providing prehospital TEE is not limited to an additional diagnostic tool; it adds at least one experienced ALS provider to the scene, which could impact the outcome of OHCA [41]. While the impact on survival is currently unknown, it can be hypothesized that early improvement in high-quality chest compression guided by TEE [7], addressing reversible causes, and increasing the safety of invasive procedures such as eCPR will prove beneficial. Cannula malposition has been reported to be around 5% in different centers [42, 43]. TEE has been demonstrated to change the management of OHCA patients between 67 and 97% [4, 9, 16]. Changes included the application of fluids or vasopressors, repositioning the AMC, immediate cath lab transfer due to regional wall motion abnormalities, or cessation of resuscitation.

Future studies should address the effect of interventions, such as AMC changes, rhythm assessment via TEE, and eCPR guidance, using prehospital TEE to close specific knowledge gaps. These studies should also plan for patient-centered outcomes such as ROSC, survival to hospital admission/ICU admission, and good neurological outcomes.

Furthermore, future studies could assess if the visual evaluation of the chest compression rate and depth with TEE is superior to an accelerometer.

## Conclusion

One observational study, two case reports, and our preliminary experience have demonstrated the feasibility of prehospital TEE. Building on this foundation, the proposed protocol offers a structured approach to integrating prehospital TEE into EMS systems. However, future work is critical to evaluate and refine this protocol further. Testing the protocol across diverse EMS systems, including urban, rural, and international contexts, will ensure its adaptability and external validity. Additionally, studies should focus on assessing the feasibility of protocol implementation, including provider training requirements, operational challenges, and patient safety considerations. By standardizing reporting and sharing findings from these studies, the protocol can serve as a valuable tool to enhance prehospital diagnostics and improve patient outcomes. Future research should also explore the clinical impact of prehospital TEE on decision-making, resource utilization, and survival rates.

## Supplementary Information

Additional file 1. Video S1 shows a mid-esophageal four-chamber view during rhythm analysis with ventricular fibrillation. Chest compressions are resumed at the end of the video before defibrillation

Additional file 2. Video S2 shows a mid-esophageal four-chamber view during chest compressions with a good area of maximal compression

Additional file 3. Video S3 shows a mid-esophageal long-axis view with an aortic valve opening during chest compressions

Additional file 4. Video S4 shows pericardial tamponade due to an aortic dissection

## Data Availability

Data sharing is not applicable to this article as no datasets were generated or analysed during the current study.

## Abbreviations

ALS: Advanced life support
AMC: Area of maximal compression
AoD SAX: Descending aorta short axis
ECMO: Extracorporeal membrane oxygenation
eCPR: Extracorporeal cardiopulmonary resuscitation
ED: Emergency department
LVOT: Left ventricular outflow tract
MELAX: Mid-esophageal long axis
ME4CH: Mid-esophageal four chamber
OHCA: Out-of-hospital cardiac arrest
ROSC: Return of spontaneous circulation
TEE: Transesophageal echocardiography
TTE: Transthoracic echocardiography
VF: Ventricular fibrillation
